# Advances in exploring the association between FMR1 premutation and fibromyalgia: a pilot study with a more effective sample definition

**DOI:** 10.1016/j.clinsp.2025.100758

**Published:** 2025-09-03

**Authors:** Mara Maria Lisboa Santana Pinheiro, Mateus Dias Antunes, Laura Machado Lara Carvalho, Anice de Campos Pássaro, Ingred Merllin Batista de Souza, Priscila de Oliveira Januário, Ariela Torres Cruz, Renan Barbosa Lemes, Frederico Monfardini, Amélia Pasqual Marques

**Affiliations:** a Department of Physiotherapy, Speech Therapy and Occupational Therapy, Faculdade de Medicina da Universidade de São Paulo (FMUSP), São Paulo, SP, Brazil; b Department of Genetics and Evolutionary Biology, Human Genetics Research Laboratory – LGH, Institute of Biosciences, Universidade de São Paulo (USP), São Paulo, SP, Brazil

**Keywords:** Fibromyalgia, Pain, FMR1, Fragile × Syndrome, Premutation, FMRP

## Abstract

**Introduction:**

The association between Fibromyalgia (FM) and the FMR1 gene premutation has been suggested, but remains inconclusively established. Previous studies often focus on whether women with FM have the FMR1 premutation, while a more appropriate approach would involve examining FM manifestation in women with the premutation. FM is a condition with multifactorial etiology, and while the rarity of the FMR1 premutation makes it unlikely that most FM cases are linked to this variation, an increased prevalence of FM among premutation carriers is still plausible. The authors present a pilot study with a more effective approach for sample definition that shows a trend toward confirming this association.

**Objective:**

To investigate the association between FM and the FMR1 premutation, using a study design that focuses on FM occurrence in women with the premutation, rather than the presence of the premutation in women with FM.

**Method:**

FM occurrence was evaluated in 140 Brazilian women aged 30 to 59, including 70 premutation carriers and 70 controls.

**Results:**

FM was diagnosed using the 2016 American College of Rheumatology (ACR) criteria. FM frequency was 25.71% among premutation carriers, compared to 12.86% in controls (*p* = 0.087). Relative risk (RR = 2) analysis showed a higher risk in the premutation group (95% Confidence Interval: 0.965‒4.143, *p* = 0.0621).

**Conclusions:**

This study employs a robust methodology with an improved sample definition and consistent diagnostic criteria. However, larger studies are needed to confirm the association and achieve statistical significance; the authors also provide a sample size calculation for future research.

## Highlights

• The preliminary data suggest an association between the FMR1 gene premutation and susceptibility to Fibromyalgia (FM), but larger studies with consistent designs are needed to confirm this hypothesis.
• A sample definition focusing on FM prevalence among premutation carriers may offer a more effective approach to exploring this association.
• The authors calculated the sample size needed to achieve statistical significance based on our pilot study.
• Investigating this association could inform future clinical management and the identification of therapeutic targets.

## Introduction

**Fig. 1:**
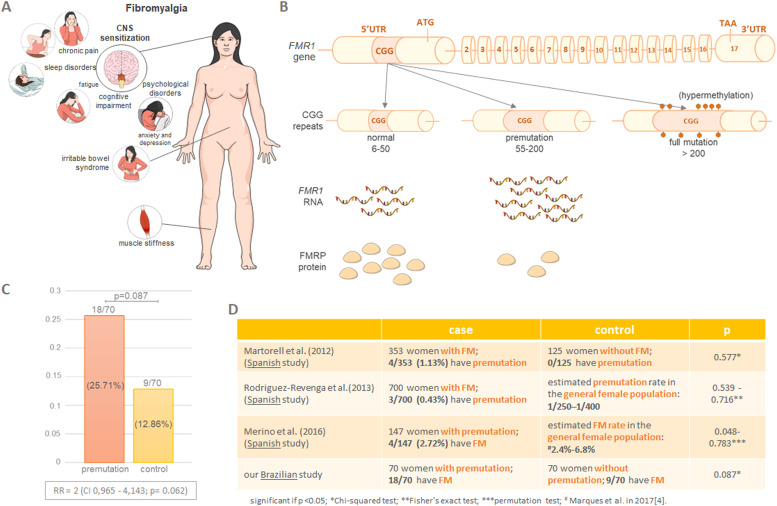
**Key clinical, molecular, and experimental insights into the study of the association between FMR1 premutation and fibromyalgia.** (A) Main clinical signs of FM. (B) The molecular consequences of CGG repeats in the FMR1 gene. The gene diagram is not to scale. (C) The main results of the pilot study. (D) Comparison of these findings with those from other studies investigating the association between FMR1 premutation and FM. Despite having a smaller sample, the experimental design resulted in a p-value closer to significance.

Fibromyalgia (FM) is a multifaceted syndrome characterized by widespread chronic pain and central sensitization of the nervous system, which often manifests alongside sleep disturbances, fatigue, and psychological alterations. FM involves both somatic and cognitive dimensions, frequently co-occurring with other functional disorders, including depression, anxiety, chronic headache, and irritable bowel syndrome^1^^,^^2^ (Fig. 1A). The complex interplay of these symptoms suggests a dysregulation of central pain processing mechanisms.^1^^,^^3^ According to a review conducted by Marques et al. in 2017, the FM rates in the general population range from 0.2 % to 6.6 %, with a higher prevalence in women (2.4 % to 6.8 %). The estimated prevalence in the general population of Brazil is 2 %.^4^

The literature suggests an increased frequency of FM in individuals with the FMR1 gene premutation, though this association is still under investigation and lacks consensus.^5^, ^6^, ^7^, ^8^, ^9^ The FMR1 gene, located on the X chromosome (Xq27.3), encodes the FMRP protein. A CGG trinucleotide repeat expansion in the 5′ UTR exceeding 200 repeats leads to hypermethylation, gene silencing, and the absence of FMRP, resulting in Fragile X Syndrome (FXS ‒ OMIM#300,624), a syndromic form of intellectual disability. A CGG repeat expansion of 55 to 200 constitutes a premutation,^10^ characterized by elevated FMR1 mRNA levels and reduced FMRP protein^11^ (Fig. 1B). This imbalance triggers ubiquitin-positive neuronal inclusions formation ‒ a pathologic hallmark of protein-mediated neurodegeneration ‒ contributing to a spectrum of clinical phenotypes known as Fragile X Premutation-Associated Conditions (FXPAC).^12^

### Hypothesis and clinical relevance of the research

This pilot study explores the hypothesis that the FMR1 gene premutation may increase susceptibility to FM. Investigating this association could provide valuable insights for clinical management, reveal underlying mechanisms, and identify new therapeutic targets for FM. Additionally, the authors provide experimental design information that will be relevant for future studies investigating this association.

### Methods

The authors evaluated 140 Brazilian women aged 30 to 59, recruited through various methods, including social media advertisements, support groups for families affected by FXS, and research institutions. The participants were divided into two experimental groups: a case group consisting of 70 women with the FMR1 gene premutation (identified through previous genetic testing, including PCR and/or Southern blotting), and a control group comprising 70 women with no positive family history of FXS or intellectual disability.

Participants were recruited between December 2020 and November 2023 through a combination of strategies, including advertisements on social media platforms, outreach in support groups for families affected by Fragile X syndrome, and contact with research institutions involved in Fragile X-related projects. Women who expressed interest in participating received a digital invitation. After providing informed consent, eligible participants completed an online questionnaire hosted on Google Forms. Inclusion criteria were: women aged 30 to 59 years, and for the case group, molecular confirmation of the FMR1 premutation by PCR and/or Southern blotting. For the control group, women with a known family history of Fragile X syndrome or with children with intellectual disability were excluded.

In both experimental groups, FM was assessed using the 2016 American College of Rheumatology (ACR) FM diagnostic criteria, which include the Widespread Pain Index (WPI) and the Symptom Severity Scale (SSS).^13^ These criteria include the Widespread Pain Index (WPI), which assesses pain in 19 specific body regions over the past week (score range: 0–19), and the Symptom Severity Scale (SSS), which quantifies fatigue, sleep disturbance, cognitive symptoms, and somatic symptoms using a 4-point Likert scale (score range: 0–12, total range: 0–31 when including somatic symptoms). To meet the diagnostic criteria for FM, participants had to meet the required combination of WPI and SSS scores, as defined in the ACR guidelines. These assessments were conducted via self-administered online forms, and instructions were provided to standardize responses (Supplementary Material 2).

In addition to the ACR diagnostic criteria, participants completed validated instruments to assess pain intensity, sleep quality, and fibromyalgia impact. Pain intensity was measured using the Visual Analogue Scale (VAS),^14^^,^^15^ where higher scores reflect greater pain severity. Sleep quality was evaluated with the Pittsburgh Sleep Quality Index (PSQI),^16^ which generates a global score ranging from 0 to 21. Fibromyalgia's impact on daily functioning and quality of life^17^ was assessed using the Fibromyalgia Impact Questionnaire (FIQ), with scores ranging from 0 to 100. These instruments were included in the online questionnaire and analyzed to complement the clinical profile of FM-affected participants in both groups (Supplementary Material 2).

This manuscript was prepared in accordance with the STROBE (Strengthening the Reporting of Observational Studies in Epidemiology) guidelines for reporting cross-sectional studies.^18^^,^^19^

## Results

### Sample characterization

Supplementary Material 1 provides the sample characterization data for all participants.

### Comparative analysis of FM prevalence in premutation carriers and controls

The authors identified a total of 18 out of 70 (25.71 %) women with FM in the premutation group, compared to 9 out of 70 (12.86 %) in the control group (p-value = 0.087 ‒ Chi-Square test; Fig. 1C). The obtained RR was 2 (95 % CI 0.965‒4.143; *p* = 0.062; see Fig. 1C).

### Sample size estimation for future studies

Given that the frequency of FM was higher in the premutation group than in controls, the authors calculated the minimum sample size required to detect a statistically significant difference under the same study design and diagnostic criteria. According to the authors’ estimation, at least 92 participants per group (a total of 184 women) would be necessary to achieve statistical significance with adequate power. This sample size calculation is provided in Supplementary Material 3 and may serve as a useful reference for future studies aiming to confirm the association between FMR1 premutation and FM.

## Discussion

Although several studies have documented a significant increase in comorbidities commonly referred to as FXPAC among carriers of the FMR1 gene premutation compared to controls, the specific association of many of these comorbidities with the premutation has not yet been firmly established. Additional studies with consistent designs are recommended to explore this relationship further, particularly in the context of chronic pain and sensitivity syndromes.

According to these results, FM is more frequent in women with the premutation than in those without, and the presence of the premutation doubles the risk of developing FM, although statistical significance was not achieved, likely due to the limited sample size available in the present study.

Interestingly, the p-value the authors obtained when comparing the case and control groups was closer to statistical significance than those reported by Martorell et al.^6^ and Rodriguez-Revenga et al.,^9^ despite the smaller sample size (Fig. 1D). This is likely due to a significant difference in sample definition. While Martorell et al.^6^ and Rodriguez-Revenga et al.^9^ investigated the presence of the premutation in women with and without FM, the authors investigated FM in women with and without the premutation.

FM has a multifactorial nature, involving complex interactions between numerous genetic and environmental factors, and has a significant prevalence (∼2 % of the general population in Brazil.^17^ The etiology and pathogenesis of fibromyalgia remain incompletely understood and are difficult to characterize due to the heterogeneous nature of its manifestations.^20^^,^^21^ FM may be comorbid with other autoimmune conditions, such as rheumatoid arthritis (25 %), systemic lupus erythematosus (30 %), and Sjögren's syndrome (50 %).^22^ Its symptoms can be exacerbated by hormonal fluctuations, physical or psychological stress, temperature changes, and alterations in diet or sleep patterns, leading to systemic effects and a significant impact on quality of life.^23^ Epigenetic changes, such as DNA hypomethylation in genes involved in stress response, DNA repair, autonomic nervous system function, and subcortical neuronal abnormalities, have been reported in individuals with FM, suggesting a complex molecular background that might contribute to susceptibility.^24^

**Fig. 2:**
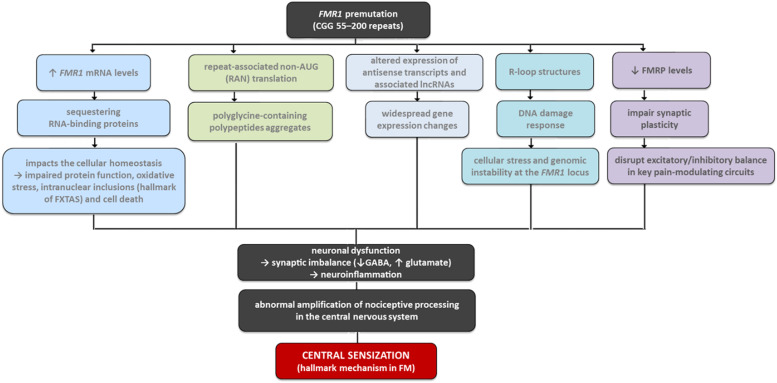
Schematic representation of potential mechanisms by which the FMR1 premutation may contribute to FM susceptibility via central sensitization.

Several biological mechanisms have been proposed to explain how the FMR1 premutation could increase susceptibility to FM (Fig. 2). One widely accepted model involves RNA toxicity due to excessive FMR1 mRNA expression. In premutation carriers, transcription is upregulated to compensate for the reduced translational efficiency of the CGG-expanded alleles.^25^ The elevated mRNA sequesters RNA-binding proteins essential for cellular homeostasis, resulting in impaired protein function, oxidative stress, and cell death.^25^^,^^26^ This process is associated with the formation of intranuclear inclusions containing misfolded proteins, which have been detected in multiple tissues, including the brain. These inclusions are a hallmark of Fragile X-associated Tremor/Ataxia Syndrome (FXTAS), a late-onset neurodegenerative disorder observed in some premutation carriers, particularly men, and characterized by intention tremor, cerebellar ataxia, Parkinsonism, and cognitive decline.^27^

In addition to this toxic RNA gain-of-function mechanism, three other pathways have been implicated in the pathophysiology of FXTAS and potentially other clinical manifestations in premutation carriers.^5^ First, repeat-associated non-AUG (RAN) translation may occur at the expanded CGG repeat, generating polyglycine-containing polypeptides that aggregate and disrupt neuronal function.^12^ Second, altered expression of antisense transcripts and associated long non-coding RNAs (lncRNAs) has been observed in post-mortem brain samples from premutation carriers. These lncRNAs are involved in chromatin remodeling and transcriptional regulation and may contribute to widespread gene expression changes.^28^^,^^29^ Third, R-loop structures ‒ RNA-DNA hybrids formed during transcription through the CGG-repeat tract ‒ can induce DNA damage and activate the DNA damage response, contributing to cellular stress and genomic instability at the FMR1 locus.^30^

These molecular alterations may interfere with neuronal signaling and stress response pathways relevant to fibromyalgia pathophysiology. In particular, central sensitization ‒ the hallmark mechanism in FM ‒ involves abnormal amplification of nociceptive processing in the central nervous system, often linked to glutamatergic hyperactivity and GABAergic inhibition deficits.^31^^,^^32^ Reduced levels of FMRP, also observed in premutation carriers, may impair synaptic plasticity and disrupt excitatory/inhibitory balance in key pain-modulating circuits,^33^^,^^34^ providing a biologically plausible link between the FMR1 premutation and enhanced pain sensitivity.

Given the rarity of the FMR1 premutation (estimated frequency of ∼1:300 women^35^), the majority of women affected by FM do not have the premutation, indicating that other factors play a predominant role in the development of this syndrome. However, this does not rule out the possibility that the FMR1 premutation may contribute to the pathophysiological mechanisms in a subset of FM cases.

As shown in Fig. 1D, the present sample’s definition appears to be more effective in evaluating the association between the FMR1 premutation and FM than the approach used by Martorell et al.^6^ and Rodriguez-Revenga et al.^9^ In contrast, Merino et al.^7^ adopted a strategy more similar to ours and reported a higher proportion of women with FM compared to the other two studies.^6^^,^^9^

The authors conducted a permutation test using the case group data from Merino et al.^7^ and the FM rate in the general female population reported by Marques et al.^17^ (2.4 % to 6.8 %). The R script adopted for this calculation is provided in Supplementary Material 3. Only the lower limit of the resulting p-value range (0.048–0.783, Fig. 1D) was statistically significant. By adopting Fisher's exact test considering the prevalence data of FM in Spanish women from Branco et al.^36^ – (27/520 or 5.2 %), the p-value was 0.27, which is not statistically significant.

The difference between the proportions of FM-positive cases in the present study and that of Merino et al.^7^ may be related to the fact that the authors investigated different populations (Brazilian and Spanish, respectively), which implies distinct genetic and environmental backgrounds. Additionally, differences in the diagnostic criteria for FM used in both studies may also have impacted the results in some way. The FM diagnosis is based on clinical symptoms, and over the years, the criteria for defining this syndrome have been modified to standardize populations for scientific studies.

As summarized in Fig. 1D, the authors provide a direct comparison between the present study and the three main investigations that previously explored this association. This study differs methodologically from those by Martorell et al. (2012) and Rodriguez-Revenga et al.,^9^ which focused on screening FM patients for the FMR1 premutation. In contrast, both the present study and that of Merino et al.^7^ investigated FM occurrence among confirmed premutation carriers. This distinction in sample definition likely explains why the p-value was closer to statistical significance, even with a smaller sample size. Taken together, these findings not only reinforce the plausibility of an association but also offer methodological insights that may help guide the design of future studies in this area.

The present findings should be interpreted as preliminary. The lack of statistical significance may be attributed to the limited sample size and the inherent heterogeneity of fibromyalgia, which is influenced by multiple genetic, environmental, and psychosocial factors. Another limitation of the present study is that no statistical adjustments were made for potential confounding variables such as age, comorbidities, medication use, or lifestyle-related factors. Moreover, as this study included only Brazilian women, the generalizability of the findings to other populations may be limited. Future studies should explore whether the observed association between the FMR1 premutation and FM holds in more ethnically and geographically diverse cohorts, which would strengthen the external validity of the results.

Finally, the authors calculated the sample size that would be necessary for the present study to achieve statistical significance, considering the same population, sample definition, and diagnostic criteria for FM. As a result, the case and control groups should each have at least 92 women. The authors believe that this information may be useful in future studies investigating the association between the FMR1 premutation and FM.

## Conclusions

This pilot study suggests a potential association between the FMR1 gene premutation and an increased susceptibility to FM. Although statistical significance was not achieved, the trends observed hint at the premutation’s potential role in FM, particularly in a subset of individuals.

The present approach ‒ focusing on FM prevalence among women with and without the premutation - provides a promising framework for further exploration. This methodology may be more effective than previous approaches. Given FM’s complex and multifactorial nature, larger studies with consistent diagnostic criteria are crucial to confirm these preliminary findings. The sample size estimates offered here can guide future research, leading to more definitive results. Future studies with larger samples should incorporate statistical adjustments for potential confounding variables using multivariate regression models or stratified analyses to better isolate the effect of the FMR1 premutation on FM susceptibility.

The authors recommend further investigation into the FMR1 premutation to advance understanding of its potential role in FM and to uncover new diagnostic and therapeutic opportunities. Considering the multifactorial nature of FM and the broader FXPAC spectrum, these findings may support more personalized approaches to clinical management and inform/ clinical practice – for example, by encouraging the screening of premutation carriers for FM symptoms.

## Consent statement/ethical approval

This study was conducted in accordance with the Declaration of Helsinki, its subsequent amendments, and the applicable legislation of the studied country. Ethical approval was obtained from the Research Ethics Committee of the Hospital Clínico da Faculdade de Medicina, Universidade de São Paulo (HCFMUSP ‒ 4.342.254). All participants provided written informed consent.

## Data sharing and data accessibility

Data Availability Statement: Retrospective data have been collected from participants over the last four years. For data requests, please contact the corresponding author. Data requests may be subject to ethical review by the Research Ethics Committee of the institution, and personal information of patients will not be disclosed to third parties under any circumstances.

## Authors’ contributions

MMLSP and APM conceptualization. MMLSP executed the methodology and wrote the first draft of this manuscript under APM's guidance. MDA and LMLC reviewed the results and assisted with manuscript writing. ACP and IMBS contributed to the development of the methodology, while POJ and ATC contributed to the interpretation of the results. FM and RBL assisted with statistical analyses. All authors read, edited, and approved the final manuscript.

## Declaration of competing interest

The authors declare no conflicts of interest.

## Appendix Supplementary materials

Image, application 1

Image, application 2

Image, application 3
