# Supplementary material for: Advances in exploring the association between FMR1 premutation and fibromyalgia: a pilot study with a more effective sample definition
Source: Clinics (Sao Paulo). 2025 Sep 3;80:100758. doi: 10.1016/j.clinsp.2025.100758 (PMC12445584; doi:10.1016/j.clinsp.2025.100758)
Supplement: Supplementary file 1 [file mmc1.docx]

**CLINICS-D-25-00188_ Supplementary Material 1**

**Supplementary Material 1**

The sociodemographic characteristics of the participants, comparing the Pre-mutation Group (PG) and the Control Group (CG), are presented in Table 1. Predominantly, both groups were white, had postgraduate education, and resided in the Southeast region of Brazil.

**Table 1** Sociodemographic data of participants.

| **Variables** | **PG (n = 70)** | **CG (n = 70)** |
| --- | --- | --- |
| **Age (mean ± SD)** | 43 ± 7.61 | 39 ± 8.44 |
| **Number of children** **(mean ± SD)** | 2 ± 0.83 | 1 ± 0.90 |
| **Race/Color** | **n (%)** | **n (%)** |
| White | 56 (80.00) | 41 (58.57) |
| Black | 1 (1.43) | 16 (22.86) |
| Brown (Mixed race) | 13 (18.57) | 10 (14.29) |
| Yellow (Asian) | ‒ | 3 (4.28) |
| **Education Level** |  |  |
| Postgraduate | 35 (50.00) | 43 (61.43) |
| Undergraduate | 18 (25.71) | 18 (25.71) |
| Elementary/High School | 17 (24.29) | 9 (12.86) |
| **Region** |  |  |
| Southeast | 45 (64.29) | 57 (81.43) |
| South | 3 (4.28) | 2 (2.86) |
| North | 3 (4.28) | ‒ |
| Northeast | 10 (14.29) | 10 (14.29) |
| Central-West | 9 (12.86) | 1 (1.43) |

GP, Pre-mutation Group; CG, Control Group; SD, Standard Deviation.

Table 2 presents the sociodemographic data of the participants in the Pre-mutation Group (PG) and the Control Group (CG) who were classified with and without Fibromyalgia (FM). Predominantly, both groups were white, pursuing postgraduate studies, and residing in the southeastern region of Brazil.

**Table 2** Sociodemographic data of participants with and without fibromyalgia in both groups (GP and CG).

| **Variables** | **GP with FM (n=18)** | **GP without FM (n=52)** | **CG with FM (n=9)** | **CG without FM (n=61)** |
| --- | --- | --- | --- | --- |
| **Age** **(mean ± SD)** | 42 ± 7.54 | 43 ± 7.61 | 45 ± 6.54 | 39 ± 8.44 |
| **Children’s number** **(mean ± SD)** | 2 ± 0.98 | 2 ± 0.83 | 1 ± 1.12 | 1 ± 0.90 |
| **Race/Ethnicity** | **n (%)** | **n (%)** | **n (%)** | **n (%)** |
| White | 15 (83.33) | 41 (78.85) | 5 (55.56) | 36 (59.01) |
| Black | ‒ | 1 (1.92) | 2 (22.22) | 14 (22.96) |
| Mixed-race | 3 (16.67) | 10 (19.23) | 2 (22.22) | 8 (13.11) |
| Asian | ‒ | ‒ | ‒ | 3 (4.92) |
| **Education** |  |  |  |  |
| Postgraduate | 10 (55.55) | 25 (48.08) | 4 (44.45) | 39 (63.93) |
| Undergraduate | 5 (27.78) | 13 (25.00) | 3 (33.33) | 15 (24.59) |
| elementary/High School | 3 (16.67) | 14 (26.92) | 2 (22.22) | 7 (11.48) |
| **Region** |  |  |  |  |
| Southeast | 12 (66.66) | 33 (63.46) | 6 (66.67) | 51 (83.61) |
| South | 1 (5.56) | 9 (17.30) | ‒ | 2 (3.28) |
| North | 1 (5.56) | 2 (3.85) | ‒ | ‒ |
| Northeast | ‒ | 3 (5.77) | 3 (33.33) | 7 (11.48) |
| Central-West | 4 (22.22) | 5 (9.62) | ‒ | 1 (1.63) |

GP, Pre-mutation Group; CG, Control Group; FM, Fibromyalgia; SD, Standard Deviation.

Table 3 presents the data of the widespread pain index, symptom severity, pain intensity, sleep quality, and fibromyalgia impact in participants diagnosed with fibromyalgia. Only pain intensity was statistically significant (p=0.033), with a higher intensity observed in the CG.

**Table 3** Clinical data of participants diagnosed with fibromyalgia.

| **Variables** | **PG (n=18) Mean ± SD** | **CG (n=9) Mean ± SD** | **p** |
| --- | --- | --- | --- |
| **WPI** (0‒19) | 8.39 ± 2.99 | 7.11 ± 2.15 | 0.190 |
| **SS** (0‒12) | 6.94 ± 1.70 | 7.67 ± 1.87 | 0.316 |
| **Pain Intensity** (0‒10) | 5.17 ± 2.46 | 6.78 ± 1.48 | 0.033^a^ |
| **Sleep Quality** (0‒21) | 10.22 ± 3.14 | 11.33 ± 3.32 | 0.365 |
| **FIQ Total Score** (0‒100) | 55.44 ± 16.17 | 62.56 ± 13.26 | 0.315 |

GP, Pre-mutation Group; CG, Control Group; SD, Standard Deviation; WPI, Widespread Pain Index; SS, Symptom Severity; FIQ, Fibromyalgia Impact Questionnaire.

^a^ Statistical significance; Nonparametric nonparametric Mann-Whitney test.; Critical level of 0.05.

No statistically significant differences were observed in the FIQ items when comparing the Pre-mutation Group (PG) and the Control Group (CG), as shown in Table 4.

**Table 4** Scores for the 10 FIQ items of participants with fibromyalgia.

| **FIQ Items** | **PG (n=18) Mean ± SD** | **CG (n=9) Mean ± SD** | **p** |
| --- | --- | --- | --- |
| **Physical Function** (0‒10) | 4.26 ± 2.41 | 4.37 ± 2.54 | 1.000 |
| **Feel Good** (0‒7) | 3.44 ± 2.12 | 3.33 ± 2.40 | 0.857 |
| **Work Missed** (0‒7) | 0.56 ± 1.15 | 0.56 ± 1.01 | 0.660 |
| **Work Ability** (0‒10) | 4.17 ± 3.60 | 6.67 ± 3.74 | 0.082 |
| **Pain** (0‒10) | 5.89 ± 3.34 | 7.67 ± 2.50 | 0.184 |
| **Fatigue** (0‒10) | 8.06 ± 2.24 | 8.33 ± 1.22 | 0.834 |
| **Morning Tiredness** (0‒10) | 6.44 ± 2.28 | 8.11 ± 1.90 | 0.099 |
| **Stiffness** (0‒10) | 6.56 ± 3.82 | 6.33 ± 3.97 | 0.857 |
| **Anxiety** (0‒10) | 7.67 ± 2.66 | 8.33 ± 2.06 | 0.660 |
| **Depression** (0‒10) | 6.72 ± 3.04 | 7.22 ± 2.64 | 0.857 |

GPF, Premutation Group; GCF, Control Group; FIQ, Fibromyalgia Impact Questionnaire; SD, Standard Deviation; significance level of 0.05; Nonparametric Mann-Whitney test; Critical level of 0.05.
