# Supplementary material for: Advances in exploring the association between FMR1 premutation and fibromyalgia: a pilot study with a more effective sample definition
Source: Clinics (Sao Paulo). 2025 Sep 3;80:100758. doi: 10.1016/j.clinsp.2025.100758 (PMC12445584; doi:10.1016/j.clinsp.2025.100758)
Supplement: Supplementary file 2 [file mmc2.docx]

**CLINICS-D-25-00188_ Supplementary Material 2**

**Supplementary Material 2**

**APPENDIX 1 – DEMOGRAPHIC AND CLINICAL DATA COLLECTION FORM**

**PATIENT'S NAME:** ______________________________________________________________________
**DATE** ____________________________________________
**AGE:** ______________
**PHONE** ______________________ / **MOBILE** ______________________
**SEX** ____________
**ID (RG)** ________________________ **Email** ______________________________________
**NATIONALITY** ______________________________________________________
**PLACE OF BIRTH** ___________________________________________________
**ADDRESS** __________________________________________________________
**RACE/COLOR:** ( ) WHITE ( ) BLACK ( ) BROWN ( ) YELLOW ( ) INDIGENOUS ( ) OTHER
**MARITAL STATUS:** ( ) SINGLE ( ) MARRIED ( ) DIVORCED ( ) WIDOWED ( ) OTHER
**OCCUPATION** ________________________________________________________
**EDUCATION LEVEL** ___________________________________________________
**WEIGHT** _____ **HEIGHT** _____
**DO YOU HAVE CHILDREN?** Yes ( ) No ( ) **NUMBER OF CHILDREN** ___
**ANY CHILD WITH A DISEASE?** Yes ( ) No ( )
**WHICH DISEASE?** ____________________________________________________
**ANY CHILD WITH INTELLECTUAL DISABILITY?** Yes ( ) No ( )

**MAIN COMPLAINT**

**PAST MEDICAL HISTORY:**

**CURRENT PAIN** Yes ( ) No ( )

**PAIN ASSESSMENT (DURATION, TYPE OF PAIN, TRIGGERING FACTORS AND FACTORS):**
**DURATION OF PAIN** __________________________________________________
**NATURE OF PAIN** ____________________________________________________

**Continuation of the Demographic and Clinical Data Collection Form**

**TRIGGERING FACTOR(S) OF THE PAIN**

**GENETIC TESTING**
**TEST LOCATION** ______________________________________________________
**TEST DATE** ___ / ___ / _________
**TYPE OF TEST** _______________________________________________________
**GENETIC TEST RESULT** ______________________________________________
**NUMBER OF CGG REPEATS** ____________________________________________

**NOTES:**

**APPENDIX 2 – WIDESPREAD PAIN INDEX (WPI)**
Mark the number of areas in which the patient has felt pain during the past week.
How many areas did the patient feel pain in? The score will range from 0 to 19.

| **Upper Left Region** | **Upper Right Region** | **Axial Region** |  |
| --- | --- | --- | --- |
| ( ) Left Jaw | ( ) Right Jaw | ( ) Cervical |  |
| ( ) Left Shoulder | ( ) Right Shoulder | ( ) Thoracic |  |
| ( ) Left Arm | ( ) Right Arm | ( ) Lumbar |  |
| ( ) Left Forearm & Hand | ( ) Right Forearm & Hand | ( ) Chest |  |
|  |  | ( ) Abdomen |  |
| **Lower Left Region** | **Lower Right Region** |  |  |
| ( ) Left Hip (buttock, trochanter) | ( ) Right Hip (buttock, trochanter) | | |
| ( ) Left Thigh | ( ) Right Thigh | | |
| ( ) Left Leg | ( ) Right Leg | | |

**Mark the areas with pain above – TOTAL POINTS (WPI):** ___

**2) Symptom Severity (SS) Scale Score**
For each of the three symptoms below, indicate the severity level during the past week, using the following scale:
 0 = no problem
 1 = mild or occasional problems
 2 = moderate, considerable problems often present and/or at a moderate level
 3 = severe: pervasive, continuous, and disruptive to daily life

- Fatigue
     Score: __
- Waking unrefreshed
     Score: __
- Cognitive symptoms
     Score: __

**In the last six months, have you experienced any of the following symptoms?**
 0 = No Problem  1 = Problem

- Headache (0–1): ___
- Abdominal pain or cramps (0–1): ___
- Depression (0–1): ___

**Continuation of the Criteria for Fibromyalgia**

**Have you experienced any of the following somatic symptoms (SS) in the past 6 months?**

( ) Muscle pain
( ) Nausea
( ) Nervousness
( ) Chest pain

( ) Blurred vision
( ) Fever
( ) Diarrhea
( ) Dry mouth
( ) Itching
( ) Shortness of breath
( ) Raynaud’s phenomenon
( ) Hives/bruising
( ) Irritable bowel syndrome
( ) Fatigue/tiredness
( ) Memory problems
( ) Muscle weakness
( ) Headache
( ) Abdominal pain/cramps
( ) Numbness/tingling
( ) Dizziness
( ) Insomnia
( ) Depression
( ) Constipation
( ) Upper abdominal pain
( ) Mouth ulcers
( ) Loss/change in taste
( ) Seizures
( ) Dry eyes
( ) Shortness of breath
( ) Loss of appetite
( ) Skin rash
( ) Sensitivity to sunlight
( ) Hearing difficulties
( ) Easy bruising
( ) Hair loss
( ) Frequent urination
( ) Painful urination
( ) Bladder spasms

**APPENDIX 3 – VISUAL ANALOG SCALE FOR PAIN (VAS)**
**Date:** ___ / ___ / ______

**Please indicate on the line below the intensity of pain you are feeling RIGHT NOW**

**0**                    **10**
|__________________________________________|
**No pain**           **unbearable pain**

**APPENDIX 4 – PITTSBURGH SLEEP QUALITY INDEX (PSQI)**

**Name:** ______________________________________________________
**Date:** ___ / ___ / ______

**Instructions:** The following questions refer to your sleep habits over the **past month only**.

1. Your answers should reflect as accurately as possible what happened **most days and nights** over the past month.
2. Please answer **all** questions.

**During the past month:**

1. **What time have you usually gone to bed at night?**
    BEDTIME: _____ : ______
2. **How long (in minutes) has it usually taken you to fall asleep each night?**
    MINUTES TO FALL ASLEEP: _____________
3. **What time have you usually gotten up in the morning?**
    WAKE TIME: _____ : ______
4. **How many hours of actual sleep do you get at night?**
   (This may be different from the number of hours you spend in bed)
    HOURS OF SLEEP PER NIGHT: _______________

**Please choose the one answer that best applies to each question. Answer all questions.**

**During the past month, how often have you had trouble sleeping because you…**

**a) Took more than 30 minutes to fall asleep**
( ) Not during the past month
( ) Less than once a week
( ) Once or twice a week
( ) Three or more times a week

**b) Woke up in the middle of the night or early morning**
( ) Not during the past month
( ) Less than once a week
( ) Once or twice a week
( ) Three or more times a week

**c) Got up to use the bathroom**
( ) Not during the past month
( ) Less than once a week
( ) Once or twice a week
( ) Three or more times a week

**Continuation of the Pittsburgh Sleep Quality Index (PSQI)**

**d) Had trouble breathing**
( ) Not during the past month
( ) Less than once a week
( ) Once or twice a week
( ) Three or more times a week

**e) Coughed or snored loudly**
( ) Not during the past month
( ) Less than once a week
( ) Once or twice a week
( ) Three or more times a week

**f) Felt too cold**
( ) Not during the past month
( ) Less than once a week
( ) Once or twice a week
( ) Three or more times a week

**g) Felt too hot**
( ) Not during the past month
( ) Less than once a week
( ) Once or twice a week
( ) Three or more times a week

**h) Had bad dreams or nightmares**
( ) Not during the past month
( ) Less than once a week
( ) Once or twice a week
( ) Three or more times a week

**i) Had pain**
( ) Not during the past month
( ) Less than once a week
( ) Once or twice a week
( ) Three or more times a week

**j) Other reason – please describe:**

**How often did this cause trouble sleeping?**
( ) Not during the past month
( ) Less than once a week
( ) Once or twice a week
( ) Three or more times a week

**Continuation of the Pittsburgh Sleep Quality Index (PSQI)**

**6) During the past month, how would you rate your overall sleep quality?**
( ) Very good
( ) Fairly good
( ) Fairly bad
( ) Very bad

**7) During the past month, did you take any medicine (prescribed, over-the-counter, or self-medicated) to help you sleep?**
( ) Not during the past month
( ) Less than once a week
( ) Once or twice a week
( ) Three or more times a week
**Which one(s)?**

**8) During the past month, how often have you had trouble staying awake while driving, eating meals, or engaging in social activity?**
( ) Not during the past month
( ) Less than once a week
( ) Once or twice a week
( ) Three or more times a week

**9) During the past month, have you had trouble with enthusiasm or energy for completing your daily activities?**
( ) No difficulty
( ) Slight difficulty
( ) Moderate difficulty
( ) Severe difficulty

**Respondent’s comments (if any):**

**Do you take naps?**
( ) No
( ) Yes

**Comments (if any):**

**Continuation of the Pittsburgh Sleep Quality Index (PSQI)**

**If yes – do you nap intentionally, that is, on purpose?**
( ) No
( ) Yes

**Comments (if any):**

**For you, napping is:**
( ) A pleasure
( ) A necessity
( ) Other – please specify: _____________________________

**Comments (if any):**

**Component Scores:**
1: _____ 2: _____ 3: _____ 4: _____ 5: _____ 6: _____ 7: _____

**APPENDIX 5 – FIBROMYALGIA IMPACT QUESTIONNAIRE (FIQ)**

**Name:** ___________________________________________________
**Date:** ___ / ___ / ______

**1. How often are you able to:**                      **Always Almost Always Sometimes Never**
a. Go shopping                                  0  1  2  3
b. Do laundry                                      0  1  2  3
c. Cook meals                                    0  1  2  3
d. Wash dishes                                   0  1  2  3
e. Clean the house (sweeping, mopping, etc.)             0  1  2  3
f. Make the bed                                 0  1  2  3
g. Walk several blocks                          0  1  2  3
h. Visit relatives or friends                        0  1  2  3
i. Take care of the yard or garden                   0  1  2  3
j. Drive a car or ride a bus                        0  1  2  3

**In the last seven days:**

**2. How many days did you feel good?**
0 1 2 3 4 5 6 7

**3. Because of fibromyalgia, how many days did you miss work (including work at home)?**
0 1 2 3 4 5 6 7

**4. How much has fibromyalgia interfered with your ability to do your job?**
☺_________________________________________________☹
**Did not interfere**              **Interfered a lot**

**5. How much pain have you felt?**
☺_________________________________________________☹
**No pain**                **A great deal of pain**

**Continuation of the Fibromyalgia Impact Questionnaire (FIQ)**

**6. Have you felt fatigued?**
☺_________________________________________________☹
**Not at all**               **Extremely**

**7. How did you feel when getting up in the morning?**
☺_________________________________________________☹
**Well-rested**                 **Very tired**

**8. Did you feel stiffness (as if your body was “locked up”)?**
☺_________________________________________________☹
**Not at all**                  **Very much**

**9. Did you feel nervous or anxious?**
☺_________________________________________________☹
**Not at all**                  **Very much**

**10. Did you feel depressed or discouraged?**
☺_________________________________________________☹
**Not at all**   **Very Much**
