# Supplementary material for: Advances in exploring the association between FMR1 premutation and fibromyalgia: a pilot study with a more effective sample definition
Source: Clinics (Sao Paulo). 2025 Sep 3;80:100758. doi: 10.1016/j.clinsp.2025.100758 (PMC12445584; doi:10.1016/j.clinsp.2025.100758)
Supplement: Supplementary file 3 [file mmc3.docx]

**CLINICS-D-25-00188_ Supplementary Material 3**

**Supplementary Material 3**

Below is the analysis script performed in R based on the case group data from Merino et al. (2016) and the same population data adopted by Rodriguez-Revenga et al. (2013). A permutation test was conducted, and as a result, a statistically significant p-value range was obtained.

case_positive <- 4

case_total <- 147

control_rate_min <- 1/400

control_rate_max <- 1/250

p_value_min <- binom.test(case_positive, case_total, p = control_rate_min, alternative = "two.sided")$p.value

p_value_max <- binom.test(case_positive, case_total, p = control_rate_max, alternative = "two.sided")$p.value

cat("P-value (min):", p_value_min)

cat("P-value (max):", p_value_max)

**p-value (min): 0.0005487439**

**p-value (max): 0.003036218**

Below is the R script used to calculate the minimum sample size required for conducting future studies, based on the same population, sample definition, and diagnostic criteria for FM, considering the data obtained from our pilot study with a Brazilian sample. As a result, the minimum sample size for each experimental group is 90.

library(pwr)
## observed data
case_total <- 70
control_total <- 70
case_positive <- 18
control_positive <- 9
## observed proportion
p_case <- case_positive / case_total
p_control <- control_positive / control_total
## size of the effect (w)
p0 <- (p_case + p_control) / 2
w <- sqrt((p_case - p_control)^2 / p0)
## test parameters
alpha <- 0.05
power <- 0.80
df <- 1
## sample size calculation
sample_size <- pwr.chisq.test(w = w, df = df, sig.level = alpha, power = power)$N
cat("Minimum sample size:", ceiling(sample_size)) **Minimum sample size: 92**
